# Scene Text Access: A Comparison of Mobile OCR Modalities for Blind Users

**DOI:** 10.1145/3301275.3302271

**Published:** 2019-03

**Authors:** Leo Neat, Ren Peng, Siyang Qin, Roberto Manduchi

**Affiliations:** UC Santa Cruz Santa Cruz, CA, USA; UC Santa Cruz Santa Cruz, CA, USA; UC Santa CruzCity, Santa Cruz, CA, USA; UC Santa Cruz Santa Cruz, CA, USA

**Keywords:** Assistive technologies, OCR, Text spotting

## Abstract

We present a study with seven blind participants using three different mobile OCR apps to find text posted in various indoor environments. The first app considered was Microsoft SeeingAI in its Short Text mode, which reads any text in sight with a minimalistic interface. The second app was Spot+OCR, a custom application that separates the task of text detection from OCR proper. Upon detection of text in the image, Spot+OCR generates a short vibration; as soon as the user stabilizes the phone, a high-resolution snapshot is taken and OCR-processed. The third app, Guided OCR, was designed to guide the user in taking several pictures in a 360° span at the maximum resolution available by the camera, with minimum overlap between pictures. Quantitative results (in terms of true positive ratios and traversal speed) were recorded. Along with the qualitative observation and outcomes from an exit survey, these results allow us to identify and assess the different strategies used by our participants, as well as the challenges of operating these systems without sight.

## 1 Introduction

A number of assistive technology application for users with visual impairment have recently appeared on the market. Powered by technological advances in AI and/or sensing infrastructure (e.g., iBeacons), these applications are designed to facilitate tasks such as wayfinding and information access without sight. In particular, SeeingAI [16], a free iOS app from Microsoft, has been met with considerable success by the blind community. SeeingAI comprises a suite of functionalities, including optical character recognition (OCR), face detection, scene description, money reader, and barcode reader. Of interest to this contribution is the ‘Short Text’ modality of SeeingAI. Short Text is a simple, powerful, and fast OCR application. It has a minimalistic user interface, which adds to its allure. Once the app is started, it simply reads aloud any text it discovers from the images continuously taken by the phone’s camera. Anecdotal evidence shows that blind users of Short Text use it for a variety of applications, such as reading the label of a bottle, accessing a sign posted at a bus stop, and reading text on a computer screen.

The ability to read text posted in the form of signs, posters, flyers, or other (sometimes called *scene text* [24]) has tremendous potential for improving information access by blind individuals. An abundance of textual signs are posted for, and routinely used by, sighted people. These include wayfinding signs, name tags, informational signs, directories, and advertising. Only a tiny portion of these signs (typically, name tags at office doors and elevator instructions) have raised characters or Braille text – and even in this case, blind people need to first locate the signs, in order to read them. Enabling blind people to read scene text would be one step towards the goal of making information accessible for all.

Automatic detection and reading of scene text on a smartphone, however, may give poor results if the user is moving around. Two main issues contribute to making this a challenging problem. First, images taken by a moving camera can be blurry, which complicates the work of OCR. Second, when exploring a scene while looking for a sign using a camera with limited field of view, it is necessary to take multiple pictures in different directions. The system needs to be able to process these images very quickly, in order to provide prompt feedback. Yet, the designer may need to trade off speed for image resolution, which is necessary for distant reading. The Short Text modality of SeeingAI, while operating at high resolution, is unable to detect text when the camera is moving. This characteristic reduces the scope of applicability of this system for scene text detection. In practice, Short Text works exceedingly well when someone knows where to aim the phone at, but may give less than satisfactory results when used to explore the scene with the camera in order to discover scene text.

**Figure 1.**
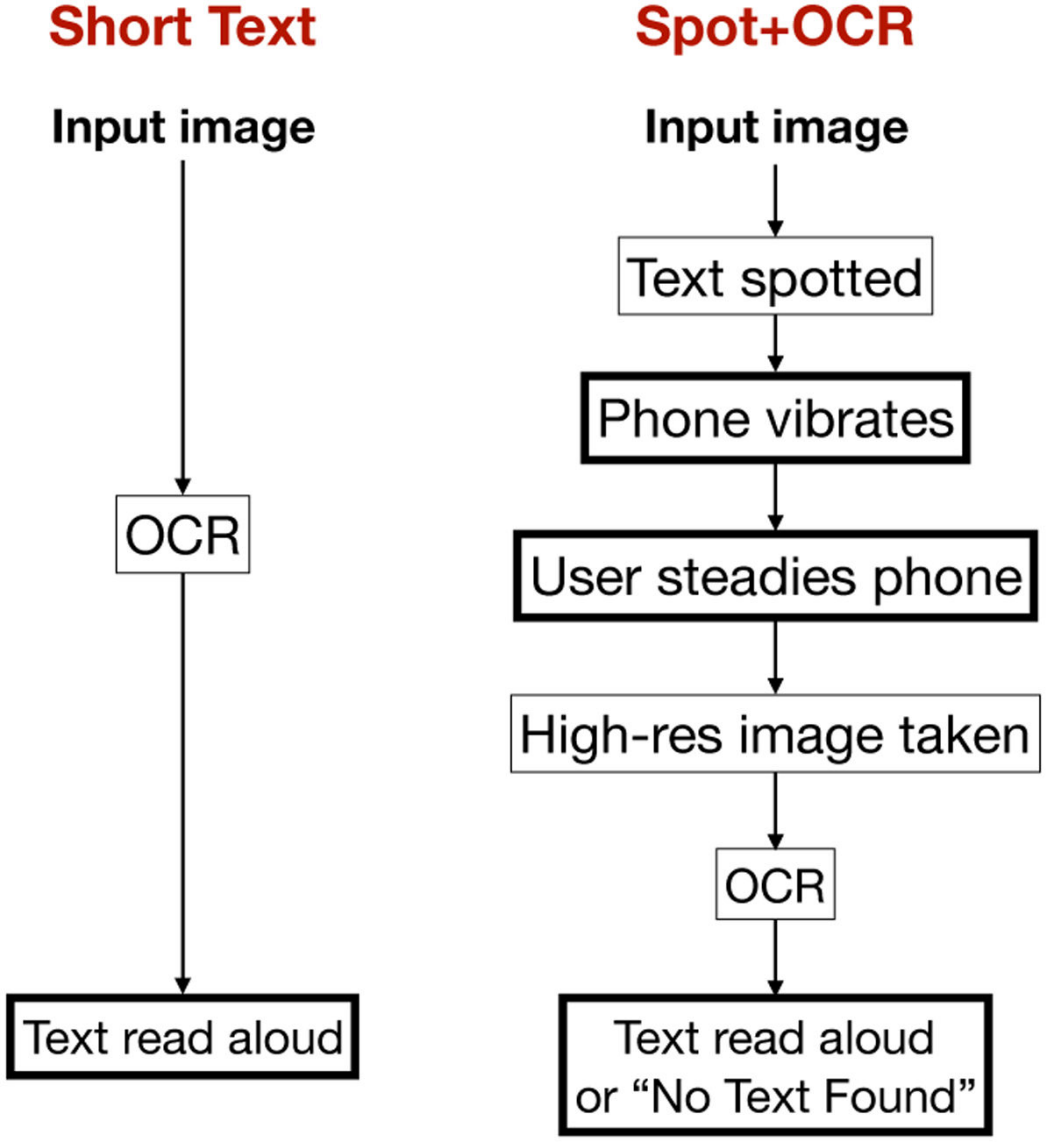
Schematic comparison of the Short Text and Spot+OCR modalities for text discovery and reading. User interface tasks are marked by a thick frame.

This paper describes an experimental comparison between Short Text and another custom system, dubbed ‘Spot+OCR’, for scene text discovery without sight. Spot+OCR, which was developed in our lab, differs from Short Text in two, interrelated aspects (see Fig. 1). First, Spot+OCR was designed to be highly sensitive, so as to discover the presence of text in an image even when taken by a moving camera. The presence of text in sight is communicated to the user via a short vibration. Second, Spot+OCR requires the user to take action, in the form of stabilizing the phone before OCR (which reads the text being detected) is activated. This is necessary because, while text can be detected even in a blurry image, *reading* the text requires a stable (and thus sharp) image. The interface modality of Spot+OCR contrasts with the minimalistic interface of Short Text, which gives no feedback to the user besides the text being read. The higher sensitivity of Spot+OCR brings on the potential for false alarms – text detected when there is none. False alarms may cause spurious vibrations, or unsuccessful reading. One goal of our experiments was to study how these two different text access modalities compare against each other when used to discover and read textual signs in realistic scenarios. More specifically, we asked whether use of Spot+OCR, with its more sensitive detection, may enable a higher rate of detection and reading of textual signs than Short Text, and whether users may find the more involved user interaction and higher rate of false alarms acceptable.

In addition to Short Text and Spot+OCR, we experimented with a third custom system (‘Guided OCR’), that we designed to search for textual signs in an open space (such as a large hall in a building). Guided OCR is an app that performs OCR on the highest image resolution afforded by the smartphone. Doing so requires substantial processing time (several seconds), and thus this system would not be suitable for exploration by continuous phone motion. Instead, the Guided OCR app guides the user in taking the minimum number of pictures with small overlapping, as necessary to cover a 360° span of the scene. Compared with Short Text and Spot+OCR, Guided OCR may afford detection and reading of textual signs from a longer distance, thus potentially reducing the need to physically explore the open space while keeping at close vicinity to its walls.

We recruited seven blind participants for a number of indoor experiments. Our participants walked along four long corridors, using Short Text or Spot+OCR to find any posted textual signs. In addition, they used all three systems in three large elevator lobbies. Quantitative measures, in the form of the rate of signs detected and correctly read, as well as the time taken for completing the tasks, were taken. Equally important, qualitative observations, along with the responses to an exit survey, highlighted the advantages and drawbacks of the systems being tested.

## 2 Related Work

The field of OCR has received renewed interest recently thanks to the development of convolutional neural network architectures, which enable accurate and robust detection and reading [21,15,19,20,25]. This technology has found its way to powerful smartphone apps such as Microsoft SeeingAI [16] or libraries such as Google Mobile Vision API [10].

It is interesting to note that the first OCR system ever demonstrated was intended for use by blind individuals [18,8]. Blind people have used OCR technology on flatbed scanners for decades. In 2005, with the release of kNFB Reader Classic, OCR was made available on smartphones [2]. Blind people quickly started using the kNFB Reader (and other mobile OCR apps) for applications other than document reading. By enabling discovering and reading of *scene text* [23] anywhere, mobile OCR apps have great potential for information access by blind people. The work described in this paper focuses on the strategies and challenges in using these systems without sight.

Prior work has addressed the issue of using a camera by blind users, with some feedback from the system to ensure that the user can frame a picture correctly [7,22,4,17]. Related work has considered access of visual information from displays appliance [11,9]. Acquiring panoramic images by blind users was considered in [26,12]. A system for guiding blind people to take OCR-readable pictures of a document with a smartphone camera was presented in [5]. Feedback from a wearable camera was used for blind walkers guidance in [6].

## 3 Method

### 3.1 Participants

Seven volunteers (three women, four men) participated in the experiment. Their age ranged between 23 and 70 (μ = 54, σ=16). All of the participants were expert independent ambulators. Two of them used a dog guide, while the remaining ones used a long cane. When walking through the corridors, one of the dog users (P1) held her dog by its harness, while the other one (P7) released the harness and held her dog by the leash. According to her, this prompted the dog to heel, rather than lead, as required for the experiment. All of the participants were blind, with at most some remaining light perception left. All of them were iPhone users. All but for P6 already had SeeingAI installed in their phone. Although a novice to SeeingAI, P6 had good experience with other OCR apps such as KNFB Reader.

### 3.2 Apparatus

#### 3.2.1 MS SeeingAI

We used the Short Text modality of SeeingAI on an iPhone SE. As mentioned earlier, Short Text simply reads aloud any text it recognizes from the images continuously taken by the camera, which is kept vertically (portrait mode). We noticed that the system also works when the camera is kept horizontally (landscape mode), however, the detection rate is much poorer in this case. If text is detected in an image, and the camera is held still, looking at the same scene, the app repeats reading the text. We noticed that in this case, Short Text often interrupts a sentence to start reading again, possibly multiple times, making it difficult at times to hear the whole sentence. If, however, one moves the camera such that the text is no longer in view, the system finishes reading the sentence without interruption.

#### 3.2.2 Spot+OCR

**Figure 2.**
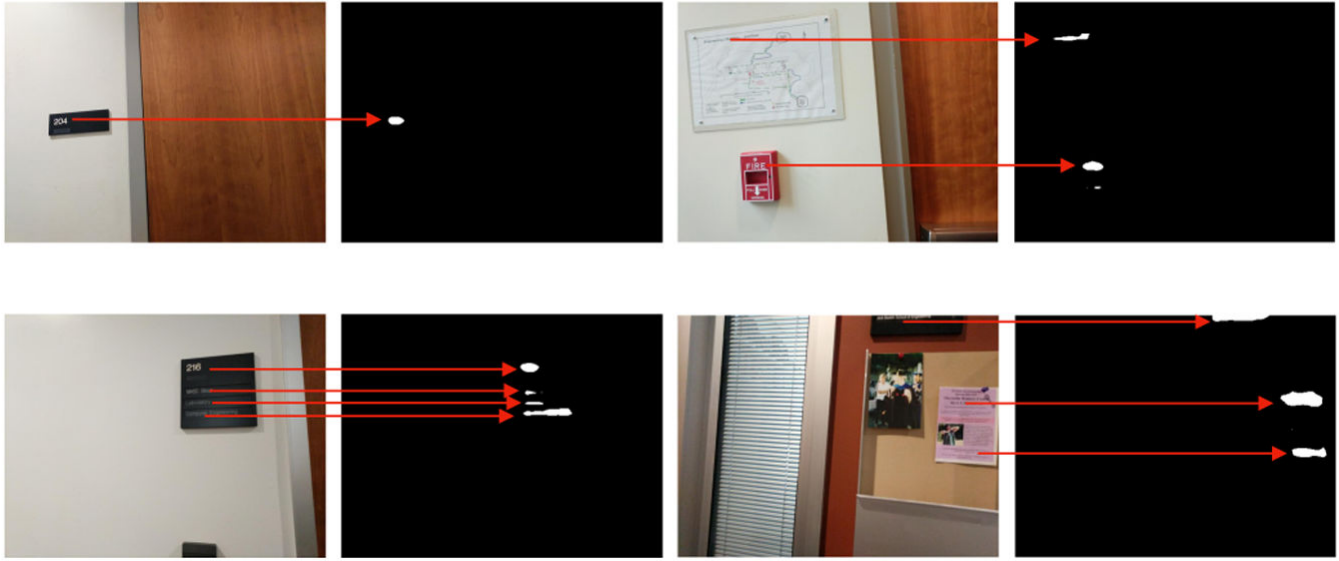
Examples of binary masks produced by the text detector from images taken by our participants in Experiment 1.

Our Spot+OCR app runs on an Android phone (we used a Google Pixel model for our experiment.) Spot+OCR requires the phone to be held horizontally (landscape mode). We found that the wider horizontal field of view (65°) increases the chance that text is detected while moving (since the same sign may be seen multiple times as one walks by or scans the scene with the camera). Spot+OCR separates the two basic functions of *detection* and *reading*. Text detection (or *spotting* [15]) is performed using a fully convolutional neural network (FCN) architecture similar to TextSegNet [19] (itself based on the FCN-8 architecture of [21]). The network was trained on images from the ICDAR 2013 [13] and the ICDAR 2015 [14] data sets. It produces a binary segmentation of the image, where ‘on’ pixels are classified as belonging to a text area. If one or more connected components with more than 100 ‘on’ pixels each are found, it is assumed that text is visible in the image. Samples of images and associated binary segmentations are shown in Fig. 2. We would like to emphasize that, while the text spotting algorithm is identical to [19], the user interface mechanism that relies on this module is a novel contribution of this paper.

Input images are first resized so that their longer side has 500 pixels. Although the network could certainly be implemented on a smartphone, achieving the desired speed (more than 1 frame per second) at the required resolution would need an engineering effort above our capacity. Instead, for the sake of the planned experiments, we opted for remote processing of the images acquired by the smartphone. Images are sent over Wi-Fi to a server workstation (3.3Ghz 6-score CPU, 32G RAM, GTX Titan X GPU and Ubuntu 14.04) using raw TCP sockets with a custom set of protocols. Once an image is captured by the Android camera, it is downsampled to 950 × 713 pixels and packaged in base64 encoding. The image is then sent to the server, unpacked, and run though the text detection network, which processes the image in about 100 ms. Depending on whether text is found, a ‘true’ or ‘false’ value is sent back to the smartphone (which does not transmit a new image to the server until a reply is received). The end-to-end delay (from the time an image is sent to the time a reply is received from the server) is of about 400 ms on average. The system is able to process about 2 images per second. Note that this remote processing strategy is liable to connection drop. In our experiments, this only occurred a couple of times, and required restarting the app. One time, the server crashed in the middle of the trial, and needed to be rebooted (causing the trial to be paused and later resumed).

Every time the smartphone system receives a ‘true’ value from the server, it is made to vibrate for 200 ms. This means that, if the camera points at a portion of text, the user feels a continuous sequence of vibrations (about two vibrations per second). If the phone is kept stable for a few seconds, a higher resolution picture (1500 × 1125 pixels) is taken for OCR processing. This event is triggered by thresholding the variance, computed over a sliding window of 5 seconds, of the magnitude of “linear” acceleration (i.e., acceleration with gravity removed) as produced by the Android SensorEvent API. The user is not notified of the high-resolution image capture.

The high-resolution image thus acquired is passed on to the Google Text Recognition API [10], which runs on the phone and produces in output any detected textual content, as well as the coordinates of axis-aligned rectangles containing the detected text areas (*text boxes*). This OCR is very accurate and fast, taking less than 400 ms to process an image. However, we observed that, similarly to Short Text, it does not have enough sensitivity to discover text when the camera is moving. This is the reason for the text spotting / user notification strategy described above.

If OCR detects text in the image, this text is read aloud using the Android TextToSpeech API. More specifically, text boxes are ordered in lexicographic order based on the position of their top left corner; then, the text within each box is read aloud one box at a time, with a 1 second delay between consecutive boxes. The text being read is prefaced by an indication of the azimuthal angle (with respect to the camera’s optical axis) of the center point of the text box, expressed in the form of clock position (e.g.: ‘At 10 o’clock: Laboratory’). If no text is found, the system utters ‘No text found’. Note that providing feedback when no text is found is important. Lacking this feedback, the user may end up holding the phone still for a long time after feeling a vibration, wondering whether a high-resolution was actually captured for OCR reading. Situations in which text is not found may occur when the text spotting system generates a false alarm, or when OCR is unable to read text (e.g. because too far). A new high-resolution image is not sent again to OCR until the system finishes reading aloud any text from the previous image, and in any case not before a 3 second interval from the previous readout.

In practice, users of Spot+OCR can explore a scene while holding the phone horizontally. If the phone vibrates, it means that there is text in sight. The user is then in charge of moving and/or orienting the phone to a position where he or she believes a good snapshot can be taken for OCR reading, then holding the phone still for a few seconds until text is read aloud or a ‘No text found’ notice is produced (see Fig. 1). Note that, due to the delay (400 ms) associated with remote processing, it may happen that the phone vibrates when the user has already passed the sign by. In our experiments, this often occurred when participants were walking along a corridor, the smartphone aimed at the wall. Upon feeling an isolated vibration, a participant can find the detected text by moving back a little, or rotating the phone in the opposite direction of walk, until the phone starts vibrating again.

A user interface provision was implemented to help users keep the phone straight. This is important because, as we observed in numerous occasions, the Google OCR API appears to produce unsatisfactory results when text is not aligned horizontally in the image. Specifically, the system utters ‘Tilt the device up/down’ if the angle between the camera’s optical axis and the horizon was found to be larger than 7.5°. Similarly, the phone utters ‘Rotate the device left/right’ if the phone was oriented around the optical axis by an amount exceeding the same threshold.

All images received by the server were stored (together with the network response), and all text produced by OCR was logged in the phone for later analysis. Unfortunately, due to a technical issue, the OCR results from the first two participants were not stored in the phone. For these trials, we used the recorded videos to carry out quantitative measurements, as discussed later.

#### 3.2.3 Guided OCR

The Guided OCR app uses the Google Text Recognition API with images at the maximum resolution produced by the camera (3840 × 2160), without a prior text detection module. It was designed to facilitate text discovery in a 360° sweep, which is the situation considered in Experiment 2. OCR processing on such high-resolution images may take up to a few seconds (depending on the content of the scene), and therefore it is important to carefully plan snapshot acquisition, lest too many overlapping images end up being processed (thus increasing the overall exploration time), or gaps be left between images (thus potentially missing textual content). Guided OCR directs the user, through a speech interface, to take a set of images at appropriate azimuth angles, where the optical axis of the camera at snapshot time forms an angle of approximately 30° with its direction in the previous image (as determined by the Android SensorEvent API).

At the beginning of a trial, once the phone is held level and stable in landscape mode, the user is prompted by the phone (via speech) to start the scan by pressing the volume button. A snapshot is taken and passed on to OCR, which then reads aloud any text found. If processing takes more than two seconds, the sentence ‘System processing’ is uttered and repeated every two seconds. After the text has been read aloud, the system prompts the user with the sentence ‘Please move slowly to the right’. (Note that the word ‘move’ here really signifies ‘rotate around a vertical axis’; however, as mentioned earlier, we already use ‘rotate’ to mean a rotation around the camera’s optical axis. In practice, only one participant got confused between the two verbs, and that was when she was asked to ‘Rotate the phone’ to adjust its orientation in an Experiment 2 trial.) The user is then expected to rotate the phone (or his/her body while holding the phone) until the correct orientation is reached, at which point the system utters ‘Stop for snapshot’. As soon as the phone is stabilized, a new picture is automatically taken and the process is repeated. If the user hasn’t reached the new position within two seconds, the phone utters ‘Please move right’ every other second. If the user overshoots the position, the system utters ‘Please move left’. Users are expected to hold the phone level during the process, in order to ensure that the space around them is explored correctly. Once the whole 360° span has been covered, the system notifies the user that the trial has terminated.

Pairs of pictures taken using this strategy have overlap of approximately 30°. While a smaller overlap could be used, we wanted to mitigate the risk of a textual sign split between two images. In case a text box is detected in each of two consecutive images within their overlapping area, and the two text boxes overlap by a large amount (as determined by homographic warping of one image into the other), only the content of the text box in the first image is read aloud.

An alternative to independent OCR processing of each acquired image could be to form and process a single panoramic image, obtained by registering and stitching all consecutive frames. This would remove any redundancy, and would also reduce the risk of text content being split between two images. However, experiments showed that the warping inherent to panoramic image stitching creates some small artifacts that sometimes hamper correct OCR reading. For this reason, we resorted to independent OCR processing of individual images, as discussed above.

### 3.3 Experiments

Each participant underwent a sequence of two experiments. In both experiments, participants used a smartphone to read posted textual signs while walking in an indoor environment. A *textual sign* could be a name or number tag posted near an office door, a flyer or a poster attached to a wall, or any other visible text, such as the writing ‘FIRE’ on an alarm pull switch. The two experiments differed in the geometry of the environments visited (see Tab. 1). All trials were video recorded. The time to complete both experiments ranged between two and three hours.

#### 3.3.1 Experiment 1: Corridors

Participants walked along two different types of corridors in our building. The first corridor type (‘Narrow’ or ‘N’) has width of 1.67 meters, with offices on one side and laboratories on the opposite side. The second corridor type (‘Wide’ or ‘W’), 2.16 meters wide, has laboratory doors on both sides. Trials were conducted in two N corridors and in two W corridors on different floors.

Before the beginning of the trials, each participant was handed the Pixel phone with the Spot+OCR app, and was instructed in the correct use of the system. Participants were given ample time to experiment with detecting and reading two office tags, and encouraged to try to approach these tags from multiple angles, in order to gain practice with the system. Participants were also shown the correct use of the Short Text modality of SeeingAI. Once a participant felt comfortable with use of both systems, Experiment 1 started.

Each participant underwent two trials with the Short Text modality in two different corridors in the same floor (one of type N and one of type W), and two trials with the Spot+OCR modality in the N and the W corridors of the other floor. The order of the modalities (Short Text, Spot+OCR) was balanced across participants, as was the order of the floors and of the corridors in a floor to be explored.

During a trial, participants walked from one end of the corridor to the opposite end, while exploring the wall on one side (of their choice) using the smartphone. Upon arriving at the end of the corridor, participants were asked to turn around and explore the other side of the corridor. The trial ended when the participant arrived back to the starting point. Participants were not asked to do anything more than discover the signs and have the phone read them aloud correctly. They were not asked to repeat or even make sense of the text uttered by the phone, nor to indicate the location of the signs or touch them. No reward was offered for discovering more signs. Participants were advised to walk in the middle of the corridor, and to experiment with different distances to the wall.

#### 3.3.2 Experiment 2: Open Spaces

Participants explored an elevator lobby with size of 5.8 × 5.8 meters. Three identical lobbies (but with different textual signs) in different floors of our building were used for this experiment. Each space was explored with a different modality (Short Text, Spot+OCR, and Guided OCR). The order of the floors and of the text reading modalities was balanced across participants, with the exception that, for the sake of simplicity, Guided OCR was always tested last. Note that Experiment 2 was conducted after Experiment 1, and by that time the participants were well acquainted with both the Short Text and the Spot+OCR modalities. Before beginning the third trial, participants were described the use of the Guided OCR app, but did not rehearse it. This was intentional, as we wanted to evaluate whether this interface modality is simple enough that it could be used “right off the box”. A trial started with the participant standing in the middle of the lobby, facing the wall opposite the elevator. Participants were tasked with finding all textual signs in the lobby, within a 360° span. They were told that they could just stand where they were and scan the environment with the smartphone, or walk around the lobby, if they so preferred. The trial ended when the participant declared that, in his or her opinion, the full span of the area had been explored.

### 3.4 Exit Interview

After completion of Experiment 2, participants were asked to take part in an interview about their experience. The interview was structured in three parts. In the first part, participants answered a questionnaire (Questionnaire 1) about their perception of the *quality of the information produced* by the system. In this questionnaire, participants were asked to not discriminate between the three different systems tested, but to comment on the general quality of the output. The goal was to probe the participants’ feelings about the general utility of OCR systems for scene text. Responses to individual statements were expressed in a Likert scale (1=don’t agree, 5=fully agree). The statements and replies to Questionnaire 1 are shown in Tab. 4. Inspired by the principles underlying the System Usability Scale (SUS [1,3]), the first eight statements alternate between “positive” and “negative”, and the statements were formulated trying to elicit strong statements of agreement or disagreement. The ninth statement is the only one directly comparing the quality of text produced by Spot+OCR and by the other systems.

Questionnaire 2 was repeated individually for each one of the three systems tested. It is composed by a subset (six) of the ten statements of the standard SUS. We decided not to use all of the SUS statements, both to reduce the load for our participants (who were asked to address 27 statements overall), and because some of the statements of SUS did not apply well to our systems (e.g. SUS statement 5, “I found the various functions in this system were well integrated”). We selected SUS statements 2, 3, 7, 8, 9, and 10. These statements alternate between “positive” and “negative”, which enables us to summarize the results using a SUS score properly normalized between 0 and 100.

The last part of the exit interview was made by a set of open-ended questions, and precisely:

Q1. In what situations do you think a system like this, that can read text in the scene, would be most useful?
Q2. Please comment on the quality of the text that was produced by the system.
Q3. Please comment on the ease of use of the system.
Q4. Any advice on how to improve the system?
Q5. Any further comments?

Participants were encouraged to specifically address individual systems in their answers to the open-ended questions.

### 3.5 Metrics

The main metric considered for quantitative evaluation of each systems was the proportion of existing text that was correctly read. Using pattern matching parlance, we call this the *True Positive Ratio (TPR)*. More precisely, the TPR is equal to the proportion of textual tokens in a “ground truth” (GT) set that were read correctly by the system (to within a certain error). We describe the creation of the GT set and the error criterion used next. The second metric was the time taken to discovered the textual signs, measured in terms of average speed in the corridors of Experiment 1, or overall time for the open spaces of Experiment 2. False alarm rates (‘No text found’ events produced by Spot+OCR) were also measured.

#### 3.5.1 Ground Truth (GT) Set

Text appears in a variety of forms in typical indoor places. The environment in which our experiments took place contained text posted as name and number tags, emergency information, floor plans, flyers and posters. Some of these signs contained a minimal amount of text (e.g. a number), while others had very long textual content (e.g. an ACM advertisement poster with a dozen of line items and more). In selecting the GT set, we decided to only keep the larger titles of posters and flyers, and to neglect smaller font content within the same sign. This decision was driven by the observation that smaller font text in these situations can only be read after the larger titles have been found, and we were more interested in the process of *discovery* of textual signs than in the accurate reading of all content in the poster or flyer. Note that situations with abundant textual content were relatively rare in the environments considered: the vast majority of textual signs only had one or a few lines of text.

The second decision we faced in the creation of the GT set was the determination of “tokens” of text, where correct discovery and reading of a token represents a successful event. Using individual words as tokens would not be very meaningful: if, say, half of the words in a sign are correctly read, the sign is probably still unintelligible. On the other hand, requiring that the whole content of the sign be read may be unnecessarily demanding. For example, consider a typical case of a sign containing a number in the first line, and a name in the second line. If only one of the two lines is read correctly, this already represents a (partial) success. Accordingly, we decided to use individual lines of text as tokens. This compromise is acceptable, in our opinion, considering that, as mentioned earlier, most signs in our building only contained one or a few lines of text.

For each environment considered, we created a catalog of text tokens, where each token was assigned an ID number. The tokens were ordered according to the prescribed traversal of the corridor from one end to the other, and back. For the open space environments, tokens were ordered according to a left-to-right sweep of the camera. In addition, tokens were clustered into groups of nearby items, such that a group of tokens could be seen from an individual picture from the camera. The reason for this clustering will be clear in the next section. We should note that one of the open space environments had one wall covered by about twenty posters with almost identical content, while another one had a wall with a large poster containing a collage of pictures and words in different languages. We decided to exclude the textual content of these walls from the GT set (even though some of these were read by the systems), as it would complicate analysis of the results. The total number of textual tokens in the environments considered is reported in Tab. 1.

#### 3.5.2 Error Criterion

The OCR apps considered generate text in the form of spoken sentences. Additionally, Spot+OCR and Guided OCR logged individual text lines (tokens). In order to decide whether one computed token matches a token in the GT set, it is necessary to determine an appropriate distance between tokens. For this purpose, we adopted the normalized Levenshtein metric [24], which is of standard use in similar situation. More precisely, two tokens are considered to be matching if their normalized Levenshtein distance is smaller than 0.2. While there is no universally agreed upon “intelligibility” threshold, a threshold of 0.2 seems appropriate for the purpose of ensuring that a token is recognizable while forgiving small editing distances. For example, the detected token ‘ybrid Systems Lal’ has distance of 0.11 to ‘Hybrid Systems Lab’ and thus is considered to be a match. However, ‘ybrid Syaaem Lal’, with a distance of 0.26, would be rejected. In practice, most words produced by the systems were either intelligible or garbled; the threshold was not found to be critical.

Another important issue is the determination of the best association between tokens read by the system (stored in a list) and tokens in the GT set. A simple solution would be to find the assignment the maximizes the total similarity (1 - normalized distance) of the matches, which amounts to computing a maximum weighted bipartite graph. This, however, would be inappropriate, for the following reason. There were many textual tokens repeating in different locations of the environments considered (e.g. the token ‘FIRE’). A successful trial would discover each one of these tokens. However, it is possible that, upon discovery of one instance of the ‘FIRE’ token, if the participant holds the camera in position, the token may be re-read multiple times. These occurrences may end up being incorrectly matched with different GT tokens (in other locations), biasing the result. Even an ordered match, where two tokens in the recorded list must have the same pairwise order as their matches in the GT set, may fail when multiple tokens are seen in the same image and read in an incorrect order. We thus resorted to the following hybrid ordered matching strategy. The list of detected tokens is first separated into sublists, where each sublist is assigned to a cluster of GT tokens, a defined in the previous section. The order of the sublists reflects the order of the groups of tokens in the GT set. Then, (unordered) maximum similarity matching is performed between tokens in a sublist and tokens in the associated GT group. After the token-level association is completed, the number of tokens in the detected list that pass the distance threshold is computed and divided by the number of tokens in the GT set to produce the TPR for that trial.

For the trials with Short Text, and for those trials using the other modalities in which data was not logged due to technical issues, the determination of TPR was based on the recorded audio of the text uttered by the system. Unfortunately, it was not possible to perfectly transcribe the text in the audio, since many of the utterances did not form proper English words (due to OCR errors producing garbled text), and letter-by-letter transcription would be exceedingly challenging in these cases. The analysis was performed as follows. First, the audio track was divided into segments, corresponding to the groups of GT tokens. Then, for each GT token in a group, the analyzer listened to the audio segment to detect a token that was perceptually similar to the GT token. If this was found, the GT token was marked as detected and removed along with the matching token. The analysis then started again with the next GT item in the group. In general, we found that detected items were either very similar or very dissimilar to corresponding GT items, and thus the risk of subjective bias in this analysis is small.

## 4 Results

### 4.1 General Observations

The participants quickly learnt to use all three modalities, including Spot+OCR and Guided OCR, which require user action in response to system feedback. The individual characters of the participants were clearly reflected in their interaction with the system. Some explored the area very methodically; upon discovery of some textual content, they would maintain or slowly vary the position of the phone, listening to the output produced, until they were satisfied that all text was correctly read. Others had a more laissez-faire attitude, and moved on after a textual sign was read just once, even if the text produced was not understandable.

In the Experiment 2 trials, most participants scanned the environment by turning around while staying in place, except for P1 and P4 who walked around the space in their first trial (both with the Short Text app), and P6 for both initial trials (with Short Text and Spot+OCR). Due to a technical issue, the Guided OCR app did not work for the trial with participant P4.

#### 4.1.1 Holding the Phone

**Figure 3.**
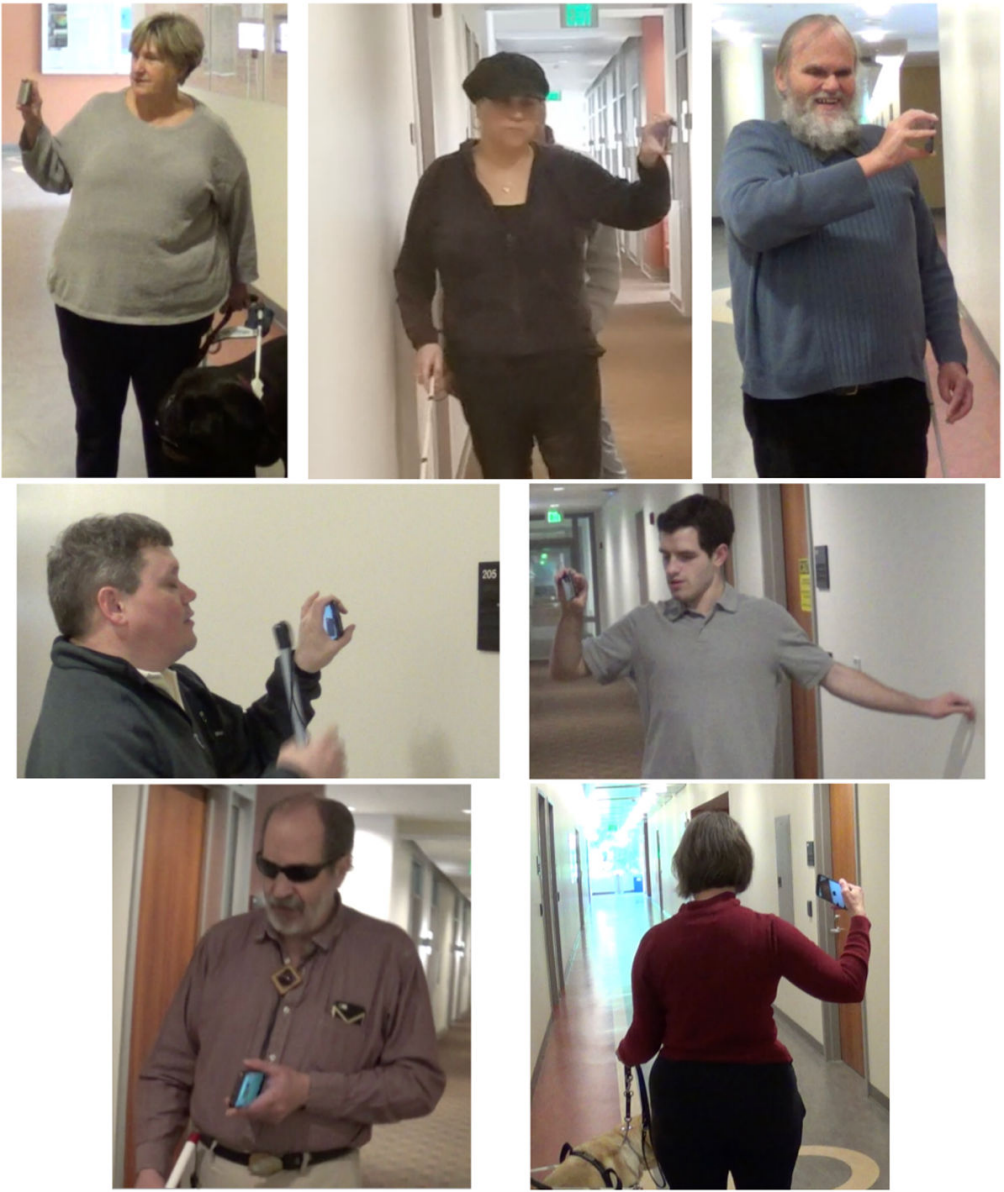
The different ways our participants held the smartphone in the Spot+OCR trials (Experiment 1)

Each participant developed his or her own strategy for holding the phone while exploring the environment (see Fig. 3). Holding the phone horizontally, which requires a pronated position of the hand, proved to be challenging for some participants. In particular, P5 needed to often stop and rest or stretch. P3 observed that, when held vertically, the phone is supported on three sides, while in a horizontal position it is supported by only two sides, which makes it less stable. In general, several participants were unable to keep the phone level, which probably contributed to less than optimal OCR reading. Remarkably, P6 found a rather efficient way to hold the phone horizontal, by resting its shorter size on his stomach. In this way, his arm wouldn’t tire, and he was able to precisely control the phone’s orientation.

#### 4.1.2 Tactile Searching

Three participants (P4, P6 and P7) explored the wall with their hand when using the Short Text app, in order to discover the location of name tags, posters and fliers, to then aim the camera at that location. Note that P4 started the trials with the Short Text app; he then would occasionally use tactile exploration even with the Spot+OCR app. P6 and P7 started with the Spot+OCR app; only in the second set of trials (with Short Text) did they decide to search for items with their hand. Note that Spot+OCR is more sensitive than Short Text, and provides vibrational feedback even when Short Text is unable to read the text. This may explain why P6 and P7 felt the need to explore the wall with their hands only when using Short Text.

### 4.2 Quantitative Results

Tabs. 2 and 3 show quantitative results in terms of TPR as well as average speed (for Experiment 1) or total time (for Experiment 2) for each trial. Speed is a more meaningful measure for Experiment 1, where participants were to walk along corridors, but would not be appropriate for the open space experiments. The average speed (in meters/minutes) was computed by dividing the total length traversed in each trial by the time taken for traversal.

#### 4.2.1 Experiment 1

The Experiment 1 data is best understood by considering that P4, P6 and P7, as mentioned earlier, explored the scene with their hand when using Short Text. By doing so, P4 and P6 achieved better TPR using Short Text than using Spot OCR. For all other participants, poorer results were obtained with Short Text. P1 was not able to find a single text token with Short Text, while P2 only found one. Better TPR values were obtained by P3 and P4, but still inferior than with Spot+OCR. It seems clear that the TPR results are dominated by whether or not the participants decided to explore the walls with their hands. Statistical analysis of this clearly bimodal data would require stratification, and thus a larger sample than used in this study to achieve adequate power. On average, the TPR rate was 27% for Short Text and 35% for Spot+OCR.

In the trials with the Spot+OCR app, ‘No text found’ notifications were produced 0.56 times as often as correctly read tokens. The ratio of garbled text (i.e., with normalized Levenshtein distance to its closest GT token in the same block larger than 0.2) to correct text tokens was 3.7. We didn’t attempt to compute this for the Short Text trials, as extracting individual garbled text tokens from the recorded audio would be exceedingly difficult.

The average velocity varied within a wide range, from 35.3 m/min for P1 with Short Text, to 2.2 m/min for P7 using Spot+OCR. On average, velocity was higher when using Short Text than when using Spot+OCR (one-tailed paired t-test, p=0.01). This may be justified by considering that Spot+OCR provided more feedback opportunities, which had to be attended to. No significant effect of corridor type was discovered on either TPR or average velocity.

#### 4.2.2 Experiment 2

The average TPR recorded were 9% (Short Text), 22% (Spot+OCR), and 45% (Guided OCR). Spot+OCR was shown by paired t-test to be associated with a TPR significantly higher on average than with Short Text (p=0.017). Guided OCR enabled higher TPR than Short Text (p=0.0016) and Spot+OCR (p=0.02). (These last two tests excluded P4, as Guided OCR did not function in his case.) The reason for the better performance of Guided OCR may be that it processes images at very high resolution, and thus can detect text from a longer distance than the other systems. No significant difference was found between the average trial time for the different modalities.

### 4.3 Exit Survey Outcome

Tabs. 4 and 5 summarize the outcomes of Questionnaire 1 and 2, respectively. While there was general consensus on some of the statements in Questionnaire 1 (e.g., S1, S2, S3), others generated disagreement (in particular, S5, S6, S9). Paired t-test shows that the average score of Questionnaire 2 for Guided OCR is significantly higher than the average score for Short Text (p=0.007), showing that our participants found Guided OCR easier to use than Short Text.

The answers to the open-ended questions brought to light a number of interesting issues. In response to Q1, participants listed multiple situations in which scene text reading would be useful, including: shopping centers, room numbers, restaurant menus, bathrooms (men/women), names on office doors, hospital room numbers, bulletin boards, signs at bus stops, train stations and airports, places with signage (e.g. laundromats), vending machines, street signs, exit signs, signs on grocery store aisles, fire instructions, hotel rooms.

Replies to Q2 were mixed, with some preferring the quality of text produced by Short Text, especially in terms of capturing the text layout (P4), and others complaining that Short Text often repeats reading a sentence before finishing it.

In replies to Q3, all except for P4 clearly stated their appreciation for the vibrational feedback provided by the Spot+OCR app. P7 observed that she didn’t mind spending time exploring a certain area if she knew that there is some text in there. This sentiment was echoed by P3, who observed that the vibrational feedback gave him the opportunity to focus on a certain direction, even if sometimes the system was unable to read the text. Interestingly, two participants mistakenly thought that the vibration rate was correlated with distance (P1) or with text centering (P5). Several participants appreciated the Guided OCR modality. In particular, P3 was enthusiastic about it, claiming that, while it may take longer to get adjusted to it (i.e. to correctly follow its directions), it may allow him to detect more text than by walking around. However, P6 complained about its relatively long processing time.

Q4 elicited several interesting suggestions, including providing more feedback for better centering of the text; the ability to take and store a snapshot when text is visible (as indicated by the vibrational feedback); providing an indication of “confidence” that text is actually there; and reducing the amount of false alarms with the Spot+OCR system. P3 wondered whether it would be possible to process the various “snippets” of text produced when trying to correctly frame the sign with the camera, to generate one coherent text sentence.

## 5 Discussion

This study has brought to light a number of interesting aspects of scene text access without sight. It is clear that, even with the best available technology, finding and reading posted textual signs is challenging for blind people. The best average TPR value obtained in Experiment 1 was just 35%; a slightly higher value (45%) was achieved in Experiment 2. This means that, on average, the participants missed, or were unable to read correctly, more than half of the textual sign tokens that were present in the environment.

In order to read a sign, it is necessary to find it first. Spot+OCR was clearly better at this than Short Text, and the participants almost universally commented very positively about the vibrational interface it offered. While the difference in detection rate could be in part attributed to the fact that the phone was held horizontally in the Spot+OCR trials and vertically for Short Text, we believe that the main reason is the increased sensitivity of the text spotting module. As mentioned earlier, Short Text simply does not read text if the camera is not kept still. Text can be also found by tactile exploration, which is how some of our participants dealt with the poor detection rate experienced with Short Text while moving the phone. P7 (who trailed the wall with her hand when using Short Text) observed, however, that tactile exploration is not always appropriate, convenient, or desirable.

Discovering text with a camera requires good awareness of the distance of the camera to where text is expected to lie. In the Experiment 1 trials, participant walked along a corridor, while aiming the camera at one of the walls. The “camera footprint”, defined as the area of the wall covered by the camera’s viewing frustum, is a direct function of the distance to the wall. Keeping far from the wall results in a large camera footprint, and thus a higher chance to detect text while moving. This is because in this case multiple consecutive pictures have overlapping footprint, and therefore the same textual sign may be seen in multiple images, thus increasing the chance that it will be detected, at least by the Spot+OCR system. This was indeed the case, and we noticed that those participants who coasted the wall opposite the one being explored seemed to achieve more detections. However, when seen from a large distance, text is acquired with low resolution, resulting in OCR reading that was often garbled, or unsuccessful. At least one participant (P3) understood this delicate trade-off and explored the wall at different distances. While this allowed him to achieve high TPR, it came at the cost of very long exploration time. Even when text is found (by the system or by touching it), framing it correctly with the camera may be difficult. Good OCR reading is achieved when text is seen head-on (fronto-parallel), at an appropriate distance. When viewed from an angle, text appears slanted (due to perspective), which complicates the job of OCR. The textual sign must be seen from a long enough distance that the whole text is within the camera’s field of view, yet close enough that it can be correctly resolved in the image. The text area must be well centered in the image, especially when it covers most of the field of view, lest part of it get cut off.

For a sighted person, who receives visual feedback from the phone’s screen, correct camera aiming is very natural. Blind users, though, may find this operation challenging. Our participants received feedback from the system, in the form of text processed by OCR and read aloud, and, for Spot+OCR, from the output of text detection (via vibration). If the output of OCR was unsatisfactory (garbled or incomplete text), participants needed to re-position or re-orient the phone to obtain a better view of it. This operation turned out to be quite difficult for most participants. Simply listening to a garbled or incomplete text sentence may not give one enough information to ascertain what the problem that caused incorrect reading may be. Is the text area seen from too large a distance? Is it seen from a bad orientation? Or was the text partially cut off from the field of view? Lacking this information, it is difficult for a user to figure out exactly where to move the camera. Some participants (in particular, P6 and P7) said that they made use of the directional information provided by Spot+OCR in the form of clock position, and rotated or moved the phone so that the text was seen at 12 o’clock. Note, however, that this solves only part of the phone orientation problem. Centering the text in the field of view does not mean that slant is removed. If one’s position is not level with the sign attached to the wall, one may need to move forward, while rotating the phone to keep the sign in sight, until the text is seen approximately fronto-parallel (i.e., the optical axis of the camera forms an orthogonal angle with the planar support of the text). This type of geometric reasoning requires clear awareness of one’s position and orientation with respect to the wall, as well as of the direction to the text, something that for a blind person may be difficult to achieve.

For those participants who used their hands to find textual signs, camera aiming was facilitated by knowing the exact location of text. Even in this case, though, we noticed that guessing the correct distance was challenging. For example, P4 kept too close to the signs discovered by tactile exploration, and for this reason sometimes only a part of a sign was read.

The problem of determining the correct location and orientation (the *pose*) of the camera for correct text framing is not unique to this application. A very similar situation occurs when taking pictures of a document laying on a desktop for OCR reading. Several systems (including the ‘Document’ mode of SeeingAI) provide feedback to the user, indicating whether the whole document is seen, or if part of it is cut off. Cutter and Manduchi [5] demonstrated a system that gives directions to the user about where to move the camera to obtain a *compliant* snapshot. The results of the study presented here suggest that a similar feedback mechanism may be beneficial also in the context of scene text reading.

Our final comment regards the Guided OCR modality. This was designed to enable accurate exploration of an open space in relatively short time. Moving around in an open space without sight is challenging, especially in an unfamiliar environment. Indeed, only three of our participants chose to explore the perimeter of the open space considered, and two of them only for one trial. Guided OCR provides the opportunity to detect text even when relatively far away (several meters of distance) while keeping in position. To do so, one must follow a fairly regimented procedure, carefully complying with guidance from the system. To our surprise, our participants did not seem to mind it, and in fact gave it the best scores in terms of usability (Questionnaire 2). Quantitatively, use of Guided OCR led to the highest TPR scores. As noted by P2, though, its use may not be desirable in public settings. In fact, the same observation may apply to the other modalities as well: other participants commented that they would be more likely to use these systems when there is no one around, lest they might attract undesired attention.

## 6 Conclusion

We have described a study with seven blind participants who explored different environments (corridors, open spaces) using three different smartphone OCR apps, in search of posted textual signs. Besides the popular SeeingAI app in its Short Text mode, the participants used two custom applications: Spot+OCR, which provides vibrational feedback when text is in sight, and then reads the text as soon as the user stabilizes the phone; and Guided OCR, which allows one to perform a 360° scan of an open place, guiding the user to correctly orient the camera, and automatically taking high resolution snapshot at the right orientations. The quantitative results, survey outcomes, and observations taken of the experiments bring to light the different strategies that blind users of these systems may adopt when searching for textual signs, and help identify the main challenges associated with use of these OCR systems without visual feedback.

## Figures and Tables

**Table 1.** Total walking length in the considered corridors (N or W type, 2^nd^ or 3^rd^ floor), and number of marked textual signs in the corridors and in the three open spaces (OS in 3^rd^, 4^th^ and 5^th^ floor).

	N2	W2	N3	W3	OS3	OS4	OS5
Tot. length (m)	85	67	85	79	—	—	—
# textual tokens	79	68	104	50	22	20	23

**Table 2:** Experiment 1 quantitative results.

Modality		Short Text		Spot+OCR	
Corridor type		N	W	N	W
**P1**	TPR (%)	0	0	28	35
	*Speed (m/min)*	*36.3*	*21.4*	*5.7*	*13.6*
**P2**	TPR (%)	1	0	32	42
	*Speed (m/min)*	*20.9*	*15.6*	*9.9*	*4.8*
**P3**	TPR (%)	18	18	38	64
	*Speed (m/min)*	*6.5*	*8.9*	*4.5*	*3.2*
**P4**	TPR (%)	63	66	35	16
	*Speed (m/min)*	*18.3*	*5.3*	*3.7*	*4.7*
**P5**	TPR (%)	12	4	23	26
	*Speed (m/min)*	*18.2*	*15.5*	*6.2*	*9.9*
**P6**	TPR (%)	69	82	46	29
	*Speed (m/min)*	*3.7*	*5.1*	*3.6*	*6.6*
**P7**	TPR (%)	11	38	31	48
	*Speed (m/min)*	*2.9*	*4.2*	*3.5*	*2.2*

**Table 3.** Experiment 2 quantitative results.

Modality		Short Text	Spot+OCR	Guided OCR
**P1**	TPR (%)	15	14	61
	*Time (s)*	*295*	*325*	*281*
**P2**	TPR (%)	4	23	50
	*Time (s)*	*233*	*594*	*443*
**P3**	TPR (%)	9	20	65
	*Time (s)*	*150*	*182*	*286*
**P4**	TPR (%)	10	17	-
	*Time (s)*	*228*	*182*	*-*
**P5**	TPR (%)	0	0	11
	*Time (s)*	*84*	*90*	*240*
**P6**	TPR (%)	27	61	55
	*Time (s)*	376	*1106*	*248*
**P7**	TPR (%)	0	17	27
	*Time (s)*	192	191	332

**Table 4.** Questionnaire 1 outcomes.

P1	P2	P3	P4	P5	P6	P7	μ	σ
S1: I thought that the text read by the systeml was very valuable								
4	4	4	5	2	4	4	3.9	0.9
S2: I found that there was too much useless information produced by the system								
2	3	**2**	1	1	3	3	2.1	0.9
S3: I think that hearing the direction towards the text was useful								
5	3	5	3	4	5	5	4.3	0.9
S4: I care only about the text content, not its precise location								
3	4	1	2	2	4	3	2.7	1.1
S5: I think that I would only use this system to find a certain desired text, rather than hearing all text in view								
4	5	1	3	2	3	2	2.9	1.3
S6: I think that is necessary to discover any text in the scene, even if not all of it is understandable								
3	1	4	1	1	3	4	2.4	1.4
S7: I think that being able to read text visible in a scene using this system would be very important in my daily life								
4	3	5	3	3	5	2	3.6	1.1
S8: I would only use this system if it worked much better than it does now								
2	2	2	1	3	4	4	2.6	1.1
S9: I felt that the text produced by the SeeingAI product was of better quality than the other systems								
1	1	1	4	2	3	4	2.3	1.4

**Table 5.** Questionnaire 2 outcomes.

P1	P2	P3	P4	P5	P6	P7	μ	σ
SeeingAI								
71	71	50	100	92	25	21	62.1	30.7
Spot+OCR								
67	83	67	67	67	50	71	67.3	9.8
Guided OCR								
92	83	71	-	87	63	96	82	12.8

## CCS Concepts

CCS → Human-centered computing → Accessibility → Empirical studies in accessibility

## References

[1] Bangor A, Kortum PT, & Miller JT (2008). An empirical evaluation of the system usability scale. Intl. Journal of Human–Computer Interaction, 24(6), 574–594.

[2] Barber M (2008). The kNFB Reader mobile: An individual perspective. Braille Monitor, 51(5).

[3] Brooke J (1996). SUS-A quick and dirty usability scale. Usability evaluation in industry, 189(194), 4–7.

[4] Bigham JP, Jayant C, Miller A, White B, & Yeh T (2010, 6). VizWiz:: LocateIt-enabling blind people to locate objects in their environment. In Proc. IEEE Workshop on Computer Vision Applications for the Visually Impaired.

[5] Cutter M, & Manduchi R (2017). Improving the accessibility of mobile OCR apps via interactive modalities. ACM Transactions on Accessible Computing (TACCESS), 10(4), 11. 29270243 10.1145/3075300 PMC5736157

[6] Fiannaca A, Apostolopoulous I, & Folmer E (2014). Headlock: A wearable navigation aid that helps blind cane users traverse large open spaces. In Proceedings of the 16th international ACM SIGACCESS conference on Computers & accessibility.

[7] Jayant C, Ji H, White S, & Bigham JP (2011, 10). Supporting blind photography In The proceedings of the 13th international ACM SIGACCESS conference on Computers and accessibility (pp. 203–210). ACM.

[8] Coughlan J, & Manduchi R (2013). Camera-based access to visual information. In Assistive technology for blindness and low vision (2013), 219–246.

[9] Fusco G, Tekin E, Ladner RE, & Coughlan JM (2014). Using computer vision to access appliance displays. In Proceedings of the 16th international ACM SIGACCESS conference on Computers & accessibility. 10.1145/2661334.2661404 PMC4269285 25531011

[10] Google Mobile Vision – Text Recognition API. https://developers.google.com/vision/android/text-overview. Last accessed 9/23/18.

[11] Guo A, Chen XA, Qi H, White S, Ghosh S, Asakawa C, & Bigham JP (2016). Vizlens: A robust and interactive screen reader for interfaces in the real world. In Proceedings of the 29th ACM Annual Symposium on User Interface Software and Technology.

[12] Hu F, Zhu Z, & Zhang J (2014). Mobile panoramic vision for assisting the blind via indexing and localization. In European Conference on Computer Vision.

[13] ICDAR Focused Scene Text Dataset http://rrc.cvc.uab.es/?ch=2. Last accessed 9/23/18.

[14] ICDAR Incidental Scene Text Dataset. http://rrc.cvc.uab.es/?ch=4&com=introduction. Last accessed 4/16/18.

[15] Jaderberg M, Vedaldi A, & Zisserman A (2014). Deep features for text spotting. In European conference on computer vision.

[16] Koul A, Li A, Haroun E, Chen IWL, Sharma S, Bianchet C, Shaikh S, Morichère-Matte S, Lai BT, Lam NPK and Lu W (2017). Augmented imaging assistance for visual impairment. U.S. Patent Application 15/242,940.

[17] Manduchi R, & Coughlan JM (2014). The last meter: Blind visual guidance to a target. In Proceedings of the ACM SIGCHI Conference on Human Factors in Computing Systems (CHI). 10.1145/2556288.2557328 PMC4241272 25426494

[18] Mann M (1949). Reading machine spells out loud. Popular Science, February 1949.

[19] Qin S, & Manduchi R (2017). Cascaded segmentation-detection networks for word-level text spotting. Proc. 14th IAPR International Conference on Document Analysis and Recognition (ICDAR 2017). 10.1109/ICDAR.2017.210 PMC5858575 29563857

[20] Shi B, Bai X, & Belongie S (2017). Detecting oriented text in natural images by linking segments. arXiv preprint arXiv:1703 06520.

[21] Tian Z, Huang W, He T, He P, & Qiao Y (2016). Detecting text in natural image with connectionist text proposal network. In European Conference on Computer Vision.

[22] Vázquez M, & Steinfeld A (2012, 10). Helping visually impaired users properly aim a camera In Proceedings of the 14th international ACM SIGACCESS conference on Computers and accessibility (pp. 95–102). ACM.

[23] Wang K, Babenko B, & Belongie S (2011). End-to-end scene text recognition. 2011 IEEE International Conference on Computer Vision (ICCV).

[24] Yujian L, & Bo L (2007). A normalized Levenshtein distance metric. IEEE Transactions on Pattern Analysis and Machine Intelligence, 29(6), pp.1091–1095. 17431306 10.1109/TPAMI.2007.1078

[25] Zhang Z, Zhang C, Shen W, Yao C, Liu W, & Bai X (2016). Multi-oriented text detection with fully convolutional networks. In Proceedings of the IEEE Conference on Computer Vision and Pattern Recognition (pp. 4159–4167).

[26] Zhong Y, Lasecki WS, Brady E, & Bigham JP (2015). Regionspeak: Quick comprehensive spatial descriptions of complex images for blind users. In Proceedings of the 33rd Annual ACM Conference on Human Factors in Computing Systems.

